# Supplementary material for: Fish can use hydrostatic pressure to determine their absolute depth
Source: Commun Biol. 2021 Oct 21;4:1208. doi: 10.1038/s42003-021-02749-z (PMC8531354; doi:10.1038/s42003-021-02749-z)
Supplement: Supplementary file 2 — Supplementary Information [file 42003_2021_2749_MOESM2_ESM.pdf]

Supplementary information: Fish can use hydrostatic pressure to determine their absolute depth.

Victoria A. Davis, Robert I. Holbrook, Theresa Burt de Perera

Supplementary methods

Study species maintenance

Subjects were captive-bred Mexican tetras, *Astyanax mexicanus* (eyed morph) between two and five years old; in the wild they inhabit freshwater habitats from the southern parts of North America to Mexico, the population used in these experiments was originally collected in Texas, North America. Individuals were raised with conspecifics in aquaria (30cm deep × 30cm wide × 91cm long) enriched with gravel and java moss. During the experiment, individuals were separated from each other by clear perforated partitions enabling fish to see and smell each other. Fish were not fed outside of the experiment unless they did not feed for more than two consecutive experimental sessions which amounted to four days, the food fish received (inside and outside of experiments) was TetraMin® tropical flakes.

Experimental setup

Apparatus

All experiments took place inside an experimental tank (90cm deep × 30cm wide × 60cm long). We covered the tank walls with white sheets to obscure the fish's views of the laboratory, leaving a small gap on one wall that allowed us to video record fish search behaviour.

Fish began all experimental trials in a 14 cm^3 start box made from clear plastic and consisting of five fixed faces, the sixth movable face formed a sliding door. We released fish from the start box by pulling clear fishing line attached to the top of the sliding door (Supplementary Figure 1); this allowed us to open the box out of view of the fish and ensured that we caused

minimal disturbance to the water in the tank. We suspended the start box from the top of the tank with fishing line, varying its height so that it was positioned either 20, 30 or 40 cm from the bottom of the tank during the experiment. This prevented fish from using the start box position as a landmark for navigation.

On the wall opposite the start box, we placed a piece of white acrylic (120cm long × 5cm wide) upon which we attached red TetraMin food flakes in a vertical line using petroleum jelly. This formed the array of flakes that the fish would have to choose from, with each flake located at a different depth. We covered all but decoy one – the edible flake, with cover slips so that these flakes were visible but inaccessible –flakes. To ensure that the absence of a cover slip was not an indicator of the position of the edible flake, we placed a cover slip directly onto the acrylic in the position of the edible flake and then placed the edible flake on top of the cover slip. The edible flake remained at a constant depth throughout all behavioural trials.

The fake floor we installed was made of opaque white acrylic and was suspended at two depths, either 17 cm or 5 cm from the bottom of the tank. We cut a small rectangular hole (2×4 cm) into the back edge of the fake floor to allow the acrylic – on which the arrays of flakes were mounted – to pass through so that it could stand on the bottom of the tank, beneath the fake floor. We suspended an acrylic ceiling from the top of the tank of similar design to the fake floor, except that there was an additional larger square hole (15 cm^2) cut into its front edge. This extra hole allowed us to lower the start box containing the fish through into the tank without having to remove the whole ceiling (Supplementary Figure 1). We then placed a lid on top of this square hole so that the fish could not see the laboratory above them from their position in the start box. As with the fake floor, we designed the ceiling so that it could be suspended from two different positions: either 63 cm or 54 cm from the bottom of the tank. Because we shifted the positions of the ceiling and the floor between

trials the total number of flakes that fish could choose from varied from between three and six on any given trial (Supplementary Figure 2).

Groups

All experimental trials took place between December 19th, 2014 and June 23rd, 2015. We divided fish into two groups that received different conditions. For the increased pressure group (n=15) the edible flake was located 45cm from the bottom of the tank and for the reduced pressure group (n=12) the edible flake was located 30cm from the bottom of the tank; group numbers were uneven as some members of the original sample of 30 had to be removed from the experiment after they became ill. Individual fish only experienced one of the two conditions; this was because the available space in the tank during the altered pressure phase of the experiment differed between the reduced pressure and the increased pressure groups. For the reduced pressure group, we removed water resulting in the functional volume of the tank becoming smaller relative to the increased pressure condition – where we added water. To ensure that the edible flake was never obscured by either the movable ceiling or floor, we positioned it at a middle depth in the tank (not close to either the top or the bottom). But because of the difference in the functional volume of the tank between the increased and reduced pressure groups we had to position the edible flake at different depths for the different groups, therefore fish could not be tested under both increased and reduced pressure conditions as their reference locations (the edible flake) were in different positions.

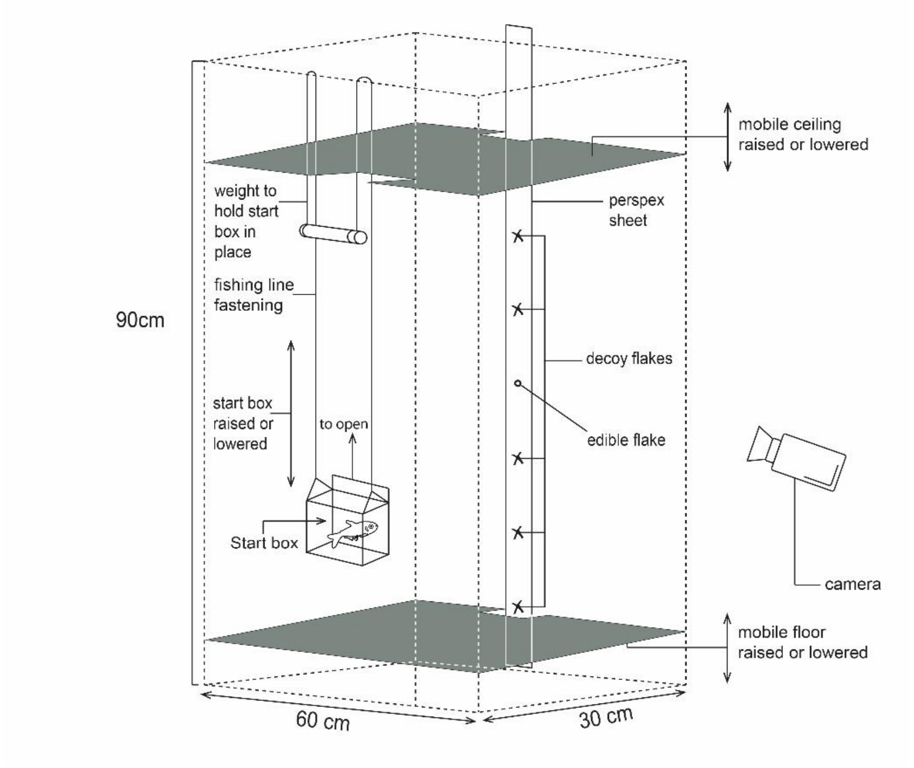

Supplementary Figure 1. Schematic diagram showing the experimental setup.

Flake arrays

We constructed three flake arrays that differed in the distance between the flakes. On array A we placed flakes 10 cm apart, on array B they were 15 cm apart and on array C they were 11.6 cm apart. This was to prevent fish from simply ‘counting’ the flakes or learning the position of the accessible flake relative to the decoy flakes. We only presented two of the three possible flake arrays to the fish. Both groups received array B but individuals in the increased pressure group additionally received array A, while those in the reduced pressure group received array C (Supplementary figure 2). Presenting identical arrays to all fish was not possible due to the limited dimensions of the tank and needing to ensure the edible flake remained at the same height for each group throughout the experiment.

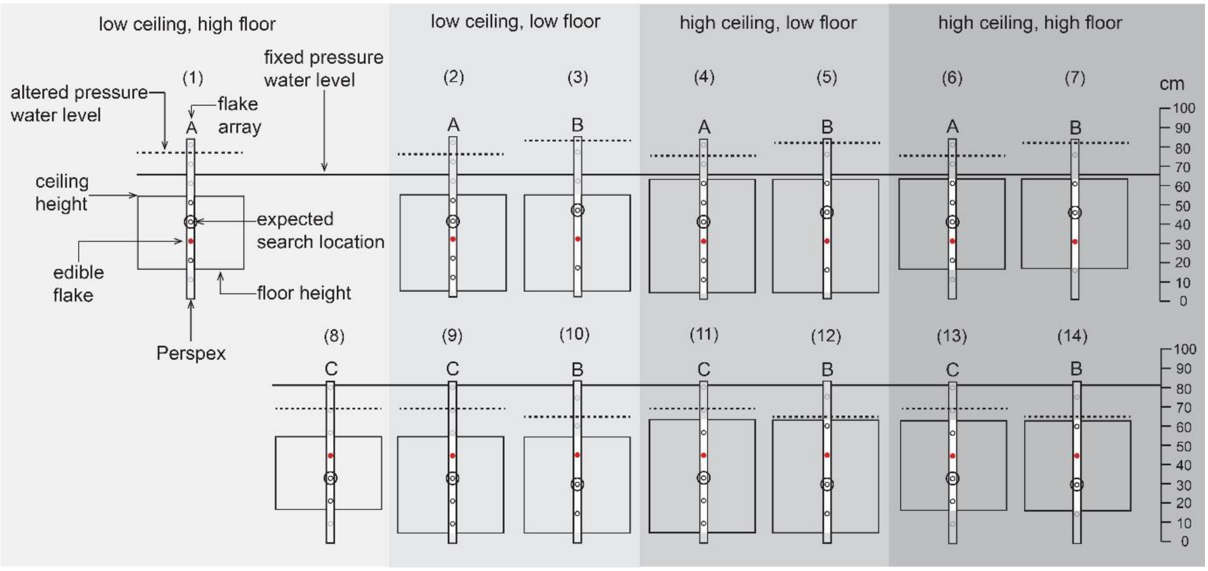

Supplementary Figure 2. Schematic diagram of the different combinations of ceiling height, floor height, and distance between food flakes in the fixed pressure phase, with the addition of the adjusted water height during the altered pressure phase**.** Fish that experienced the reduced pressure condition were presented with the combinations on the top row (1-7) and fish that experienced the increased pressure condition were presented with the combinations on the bottom row (8-14). The top and bottom of the boxes mark the depths of the floor and ceiling (fish could search anywhere between these – inside the bounds of the rectangle). The letters refer to the three flake arrays (A, B or C). The small open circles represent the decoy flakes (covered with cover slips), the red circle represents the edible flake, and the large circle highlights the flake we predicted the fish to select during the altered pressure phase if they responded according to the pressure change that we imposed. The long solid line along the top of all the tank combinations represents the level of the water during the fixed pressure phase of the experiment and the dotted lines represent the level of the water during the altered pressure phase of the experiment.

Experimental procedure

Fixed pressure phase

Fish in the increased pressure group experienced a water depth of 66 cm during the fixed pressure phase, whilst the fish in the reduced pressure group experienced a water depth of 81 cm. We allowed fish 60s to settle in the start box, after this time we raised the door and gave them 600s to select a flake. We ended the trial once the fish had chosen a flake or after 600s had elapsed. We considered the first flake that the fish's mouth touched to be the fish's 'choice'. If the fish chose the edible flake, we allowed it 120s to consume it and considered it to have passed that trial. If the fish chose one of the decoy flakes we considered it to have failed that trial and allowed it to continue its search for the edible flake for the remainder of the trial. Fish moved on to the altered pressure phase of the experiment once they had passed the learning criterion – that is, they had chosen the edible flake on their first attempt in nine of the previous ten trials. We removed any fish from the experiment that failed to reach the criterion within 100 trials and placed them into separate aquaria. Between altered pressure trials we continued fish training, setting the subsequent pass criterion at four out of the previous five trials, to ensure that they continued to remember the location of the edible flake during the subsequent trials.

Altered pressure phase

During the altered pressure phase of the experiment, we added water in trials with the increased pressure group and removed water in trials with the reduced pressure group. We covered the previously edible flake with a cover slip so that all flakes were decoys. This was to uncover any individuals that had learnt the vertical location of the food using cues other than hydrostatic pressure. For example, if fish searched in the same location as they had searched during the fixed pressure phase, this would have indicated that fish were using external visual landmarks that could be seen from the side of the tank where we placed the

camera to record trials. Similarly, if fish had used differences in olfactory cues emanating from the edible flake during the fixed pressure phase, we would expect them to search randomly during the altered pressure phase, since all flakes were covered during this part of the experiment and thus no differences in the strength of olfactory cues would have been detectable. Only if fish were using hydrostatic pressure to identify the location of the food site would we expect to observe fish shifting their search for the flake in the direction that the pressure cues dictated: searching at shallower depths after an increase in pressure and at deeper depths after a decrease in pressure (Supplementary Figure 3). Altered pressure tests were performed immediately after fixed pressure tests were over to ensure that the swim bladder was primed to detect the pressure change, as over longer time scales, the swim-bladder would have adjusted (either by inflating or deflating) to allow the fish to maintain neutral buoyancy, and thus prevented fish from deriving an absolute sense of hydrostatic pressure from the swim bladder.

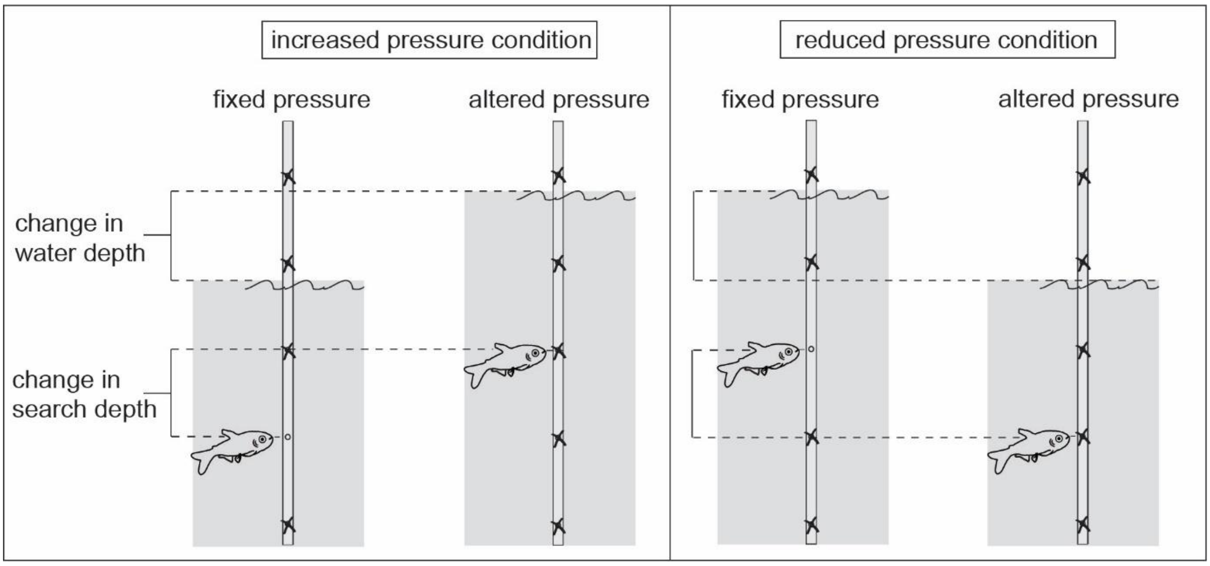

Supplementary Figure 3. Predicted shifts in search location between fixed and altered pressure trials after water has been added (increased pressure condition) or removed (decreased pressure condition). The small circle indicates the edible flake and the crosses represent the decoy flakes.

Altering pressure

The amount of water we added to or removed from the tank corresponded with the between-flake distances of the flake array that was presented to the fish on that particular trial. For example, if we presented fish with array B where flakes were 15cm apart we removed (or added) 15cm of water, this was so that there would always be a flake in the location we predicted fish would search after the pressure was altered, as we knew fish would not search in an area where they could see that there was no food. Because we gave the fish three trials but only two flake arrays, we presented each fish with one array once and the other array twice. We balanced this across fish, where possible, so that approximately one third received array A twice, a third received B twice and a third received C twice.

Permutation tests

Fixed pressure phase

To test whether the number of fish that passed the learning criterion during the fixed pressure phase was greater than we would have expected by chance, we permuted the behaviour of fish searching randomly and compared the proportion of 'random' fish that found the edible flake first time with the proportion of real fish that we observed to find the edible flake first time. To create the random data, we generated random flake choices, in which a single flake choice represented the result of one behavioural trial. The randomly behaving fish would choose any of the flakes with equal probability (1/f, where f = number of flakes available during a trial). Therefore, the probability of choosing the accessible flake depended on the number of flakes that the random fish had to choose from on any given trial. We generated 27 sets of flake choices, one for each of the fish that took part in the fixed pressure phase. Both the number of flake choices generated (corresponding to the number of trials) and the probability of picking a certain flake (total number of flakes) that random fish received were

identical to those experienced by the real fish. Based on the 27 random fish choices, we calculated how many of these random fish passed criterion (i.e. located the accessible flake on their first attempt in nine out of any ten consecutive trials). We repeated this process 1000 times, giving 1000 permutations of the total number of fish to find the edible flake during the fixed pressure phase (in each case, this number could range between 0, indicating that none of the random fish found the edible flake, and 27, indicating that all of the random fish found the edible flake). We then calculated the p-values as the proportion of these 1000 permutations in which the number of random fish to pass the criterion was equal to or greater than the number of fish to do so in our real data (=10 out of the 27 fish).

Altered pressure phase

Ten individuals successfully learnt the position of the edible flake in the fixed pressure phase and were thus included in the altered pressure phase. To test whether the total number of trials (three trials per each of the ten fish) where the predicted flake choice (according to pressure) was chosen more frequently than we would have expected by chance, we tested the probability of finding our result if the fish were behaving in the altered pressure phase as they had during the fixed pressure phase of the experiment. Unlike the random null choices, the fixed pressure null choices also accounted for differences in search behaviour between individuals. To generate the fixed pressure null expectation, we matched each of the 30 trials with fixed pressure trials whose conditions were identical to those of each altered pressure trial, based on three criteria: floor depth, ceiling depth and flake array. Matching the altered pressure trials with the fixed pressure trials using these criteria ensured that each subset of fixed pressure trials was exactly equivalent to the altered pressure trial, except that there was no change in pressure. Matching the trials in this way also meant that for each altered pressure trial, we had comparable fixed pressure trials where fish had an identical number of flake options to choose from in both experimental phases. Once we had matched each altered

pressure trial with a subset of the fixed pressure trials, we randomly drew a flake chosen by each fish during one of the matched fixed pressure trials and treated that as the flake chosen in the altered pressure trial. We did this for all 30 of the trials and calculated the number of these trials that were passed, i.e., in which the fish chose the flake we predicted according to the change in hydrostatic pressure.

Using each of the random and fixed pressure null models for flake choice, we permuted the flake choices for all 30 trials 1000 times, which gave 1000 pass counts (ranging between 0 and 30 in each case). We then calculated the p-values as the proportions of the 1000 permutations in which the total number passed trials (i.e., in which the expected flake was chosen) was greater than or equal to the observed number of passed trials in our real data (=17 out of the 30 trials).
