# Supplementary material for: Fish can use hydrostatic pressure to determine their absolute depth
Source: Commun Biol. 2021 Oct 21;4:1208. doi: 10.1038/s42003-021-02749-z (PMC8531354; doi:10.1038/s42003-021-02749-z)
Supplement: Supplementary file 3 — Description of Additional Supplementary Files [file 42003_2021_2749_MOESM3_ESM.pdf]

Description of Additional Supplementary Files

File name: Supplementary Data 1.
Description: Source data used to generate Fig. 2a.
