# Supplementary material for: Fish can use hydrostatic pressure to determine their absolute depth
Source: Commun Biol. 2021 Oct 21;4:1208. doi: 10.1038/s42003-021-02749-z (PMC8531354; doi:10.1038/s42003-021-02749-z)
Supplement: Supplementary file 5 — Reporting Summary [file 42003_2021_2749_MOESM5_ESM.pdf]

nature research

Corresponding author(s): Victoria A. Davis
Theresa Burt de Perera

Last updated by author(s): Aug 18, 2021

Reporting Summary

Nature Research wishes to improve the reproducibility of the work that we publish. This form provides structure for consistency and transparency in reporting. For further information on Nature Research policies, see our Editorial Policies and the Editorial Policy Checklist.

Statistics

For all statistical analyses, confirm that the following items are present in the figure legend, table legend, main text, or Methods section.

n/a	Confirmed	
☐	☒	The exact sample size (n) for each experimental group/condition, given as a discrete number and unit of measurement
☐	☒	A statement on whether measurements were taken from distinct samples or whether the same sample was measured repeatedly
☐	☒	The statistical test(s) used AND whether they are one- or two-sided<br>*Only common tests should be described solely by name; describe more complex techniques in the Methods section.*
☐	☒	A description of all covariates tested
☐	☒	A description of any assumptions or corrections, such as tests of normality and adjustment for multiple comparisons
☐	☒	A full description of the statistical parameters including central tendency (e.g. means) or other basic estimates (e.g. regression coefficient) AND variation (e.g. standard deviation) or associated estimates of uncertainty (e.g. confidence intervals)
☐	☒	For null hypothesis testing, the test statistic (e.g. F, t, r) with confidence intervals, effect sizes, degrees of freedom and P value noted<br>*Give P values as exact values whenever suitable.*
☒	☐	For Bayesian analysis, information on the choice of priors and Markov chain Monte Carlo settings
☒	☐	For hierarchical and complex designs, identification of the appropriate level for tests and full reporting of outcomes
☒	☐	Estimates of effect sizes (e.g. Cohen's d, Pearson's r), indicating how they were calculated

Our web collection on statistics for biologists contains articles on many of the points above.

Software and code

Policy information about availability of computer code

Data collection	No software or code was used for data collection
Data analysis	R version 3.3.3

For manuscripts utilizing custom algorithms or software that are central to the research but not yet described in published literature, software must be made available to editors and reviewers. We strongly encourage code deposition in a community repository (e.g. GitHub). See the Nature Research guidelines for submitting code & software for further information.

Data

Policy information about availability of data

All manuscripts must include a data availability statement. This statement should provide the following information, where applicable:

- Accession codes, unique identifiers, or web links for publicly available datasets
- A list of figures that have associated raw data
- A description of any restrictions on data availability

Data available on request through contacting the corresponding authors

Field-specific reporting

Please select the one below that is the best fit for your research. If you are not sure, read the appropriate sections before making your selection.

☐ Life sciences ☒ Behavioural & social sciences ☐ Ecological, evolutionary & environmental sciences

For a reference copy of the document with all sections, see nature.com/documents/nr-reporting-summary-flat.pdf

Behavioural & social sciences study design

All studies must disclose on these points even when the disclosure is negative.

Study description	Observational study collecting quantitative data on the depth that fish search for food before and after a change in water pressure.
Research sample	27 Captive-bred Mexican tetras, Astyanax mexicanus (eyed morph) between two and five years old, subspecies originally from Pachón caves in Mexico, sex unkown. We chose this species after extensive previous experiments demonstrated that they could navigate vertically in the absence of visual and olfactory cues. We hypothesised they were using hydrostatic pressure for navigation and wanted to test this.
Sampling strategy	We used a random sampling technique to select fish. We used previous studies on that we published on this species to inform the sample size.
Data collection	The researcher used video equipment to record the fish search depth and then recorded the results using pen and paper. The researcher was not blind to the study hypothesis or the experimental conditions.
Timing	19/12/2014 - 23/06/2015 - experiments took place daily over this period.
Data exclusions	Three fish were removed from the experiment as they became ill. The remaining Fish moved on to the altered pressure phase of the experiment once they had passed the learning criterion – that is, they located the food on their first attempt in nine of the previous ten trials. We removed any fish from the experiment that failed to reach the criterion within 100 trials and placed them into separate aquaria. Between the altered pressure trials the subsequent pass criterion at four out of the previous five trials.
Non-participation	Three fish were removed after they became ill, and 17 fish did not get tested under the altered pressure phase as they did not pass criterion.
Randomization	Fish were allocated to one of two groups, either the group that experienced an increase in pressure or the group that experienced a decrease in pressure. They were allocated randomly, each was numbered and all the odd numbered fish were in the increased pressure group and the even fish were in the decreased pressure group.

Reporting for specific materials, systems and methods

We require information from authors about some types of materials, experimental systems and methods used in many studies. Here, indicate whether each material, system or method listed is relevant to your study. If you are not sure if a list item applies to your research, read the appropriate section before selecting a response.

Materials & experimental systems

n/a	Involved in the study	
☒	☐	Antibodies
☒	☐	Eukaryotic cell lines
☒	☐	Palaeontology and archaeology
☐	☒	Animals and other organisms
☒	☐	Human research participants
☒	☐	Clinical data
☒	☐	Dual use research of concern

Methods

n/a	Involved in the study	
☒	☐	ChIP-seq
☒	☐	Flow cytometry
☒	☐	MRI-based neuroimaging

Animals and other organisms

Policy information about studies involving animals; ARRIVE guidelines recommended for reporting animal research

Laboratory animals	Captive-bred Mexican tetras, Astyanax mexicanus (eyed morph) between two and five years old, subspecies originally from Pachón caves in Mexico, sex unkown.
Wild animals	Study did not involve wild animals
Field-collected samples	Study did not involve samples collected from the field

Ethics oversight	Ethical approval was given by Zoology AWERB (Animal Welfare and Ethics Review Board) and the work did not require a Home Office Licence.

Note that full information on the approval of the study protocol must also be provided in the manuscript.
